# Green Extraction of Phenolic Compounds from Blueberry (*Vaccinium corymbosum* L.) By-Products Using Natural Deep Eutectic Solvents for Cosmetic Applications ^†^

**DOI:** 10.3390/antiox15060744

**Published:** 2026-06-11

**Authors:** Yassine Jaouhari, Giuseppe Morreale, Lorella Giovannelli, Elia Bari, Hélder Oliveira, Nuno Mateus, Alessandro Candiani, Beatriz Gullón, Matteo Bordiga, Jean Daniel Coïsson, Pedro Ferreira-Santos

**Affiliations:** 1 Dipartimento di Scienze del Farmaco, Università Degli Studi del Piemonte Orientale “A. Avogadro”, Largo Donegani 2, 28100 Novara, Italy; yassine.jaouhari@uniupo.it (Y.J.); 20032555@studenti.uniupo.it (G.M.); elia.bari@uniupo.it (E.B.); alessandro.candiani@uniupo.it (A.C.); matteo.bordiga@uniupo.it (M.B.); jeandaniel.coisson@uniupo.it (J.D.C.); 2 nutractiva S.r.l., Largo Donegani 2, 28100 Novara, Italy; 3 Associated Laboratory for Green Chemistry (LAQV) of the Network of Chemistry and Technology (REQUIMTE), Department of Chemistry and Biochemistry, Faculty of Sciences, University of Porto, 4169-007 Porto, Portugal; helder.oliveira@fc.up.pt (H.O.); nbmateus@fc.up.pt (N.M.); 4 Department of Chemical Engineering, Faculty of Science, University of Vigo (Campus Ourense), As Lagoas, 32004 Ourense, Spain; bgullon@uvigo.gal (B.G.); pedrosantos@ceb.uminho.pt (P.F.-S.); 5 IAA—Instituto de Agroecoloxía e Alimentación, University of Vigo (Campus Auga), As Lagoas, 32004 Ourense, Spain

**Keywords:** NaDES, antioxidant, berry by-products, sustainability, antiaging properties, cosmetics

## Abstract

The valorization of agri-food by-products generated during juice extraction represents a key strategy within circular economy frameworks, as it reduces the environmental impact of waste disposal while creating added value and improving the food supply chain. In this work, five betaine-based natural deep eutectic solvents (NaDES) differing in their hydrogen-bond donors, namely citric acid, lactic acid, acetic acid, glycerol, and ethylene glycol, were used for the green extraction of blueberry pomace, a largely underutilized by-product that is nevertheless rich in bioactive compounds. The extracts were characterized by liquid chromatography coupled with diode-array and tandem mass spectrometric detection, allowing targeted profiling of anthocyanins and non-anthocyanin phenolics, including phenolic acids, flavonoids, and phenolic aldehydes. The extraction performance of NaDES was benchmarked against conventional solvents (water and ethanol) to evaluate differences in selectivity and efficiency toward distinct phenolic classes. Antioxidant capacity was determined using DPPH and ABTS radical scavenging assays. Among the NaDES systems, the betaine–citric acid NaDES extract exhibited notable phenolic recovery together with marked radical scavenging activity. After evaluating its inhibitory activity against elastase and tyrosinase, enzymes involved in the skin aging process, the selected NaDES extract was incorporated into a natural-based antiaging cosmetic formulation, and its main physicochemical properties were assessed to verify suitability for topical application. This study demonstrated that the use of NaDES represents an environmentally friendly and sustainable approach to transform blueberry by-products into high-value, safe, and ready-to-use cosmetic functional ingredients without the need for solvent removal.

## 1. Introduction

The exponential growth of the global population, which reached eight billion in 2022, has intensified the focus on sustainable development, as formalized by the 17 Sustainable Development Goals (SDGs) established by the United Nations in 2015 [1]. Among these, SDG-12 aims to reduce food waste and loss by 50% by 2030 and represents a key strategy for promoting sustainable consumption and production patterns. Within this framework, the agri-food industry constitutes one of the major contributors to food waste, generating large quantities of by-products that are often discarded despite their high added-value potential [2]. Within the agri-food sector, the juice industry generates substantial amounts of pomace, a by-product composed of fruit skins, seeds, and pulp remaining after juice extraction. Blueberry pomace (BP), in particular, is recognized as a rich source of phenolic compounds, including anthocyanins and phenolic acids [3,4]. These compounds exhibit well-documented antioxidant, anti-inflammatory, and antimicrobial properties, making them attractive candidates for applications in nutraceuticals, cosmetics, and pharmaceutical fields [5,6,7]. In cosmetic applications, phenolic compounds contribute to skin protection not only through their antioxidant and antiradical activity but also by modulating biological pathways involved in skin aging [8,9]. Furthermore, among extrinsic aging factors, ultraviolet (UV) radiation is recognized as the primary driver of premature skin aging [10], mainly due to excessive production of reactive oxygen species (ROS) and the resulting oxidative stress in epidermal tissues. Anthocyanins, which are abundantly present in BP, have demonstrated photo-chemoprotective effects against UV-B-induced skin damage. For example, topical application of extracts rich in cyanidin-3-*O*-sophoroside and cyanidin-3-*O*-sambubioside has been shown to reduce oxidative stress and apoptosis in mouse skin tissues, potentially through activation of the nuclear factor erythroid 2-related factor 2 (Nrf-2) pathway and its interaction with mitogen-activated protein kinase (MAPK) and nuclear factor-κB (NF-κB) signaling pathways [11,12,13]. Beyond direct oxidative damage, ROS accumulation can also stimulate the activity of dermal enzymes such as collagenase, elastase, and hyaluronidase, which degrade collagen, elastin, and hyaluronic acid—key components responsible for maintaining skin firmness, elasticity, and structural integrity. Phenolic-rich extracts have been reported to inhibit these enzymes through direct interaction and complex formation, thereby contributing to the mitigation of skin aging processes [14,15].

Despite the strong interest in phenolic extracts from berry pomace, their industrial exploitation is constrained by conventional extraction methods, which commonly employ organic solvents such as methanol, ethanol, or acetone. These solvents raise environmental and safety concerns and often require solvent-removal steps before formulation [16]. As a result, growing emphasis on sustainability and green chemistry has driven the development of alternative extraction strategies that are both environmentally friendly and economically viable [17,18]. Among emerging green solvents, Deep Eutectic Solvents (DES), particularly Natural Deep Eutectic Solvents (NaDES), have gained attention as effective alternatives. NaDES consist of two or more natural and biodegradable components acting as hydrogen bond acceptors (HBAs) and donors (HBDs), which interact through hydrogen bonding to form liquids with melting points lower than those of their individual constituents [19,20]. Their non-toxic, non-flammable, and recyclable nature, combined with the possibility of tailoring their composition for selective extraction, makes NaDES particularly suitable for green recovery of bioactive compounds. Importantly, NaDES do not require solvent removal and can serve simultaneously as extraction media and formulation components, making them “ready-to-use” systems. This aspect is particularly relevant for cosmetic applications, where NaDES have been proposed not only as extraction media but also as bio-based matrices for obtaining natural antioxidant-enriched ingredients directly applicable in formulations. The application of NaDES for the valorization of BP is therefore consistent with circular economy principles, enabling the conversion of agri-food by-products into high-value functional ingredients for cosmetic and skincare applications [21]. Among potential hydrogen bond acceptors, betaine has emerged as a particularly attractive component for NaDES formulation due to its biodegradability, low toxicity, and regulatory approval for cosmetic use within the European Union, in contrast to choline, which is listed in Annex II of the European Regulation on Cosmetic Products [22,23].

Although the use of betaine-based NaDES for plant polyphenol extraction and the recovery of phenolic compounds from blueberry by-products have been previously investigated, most available studies have mainly focused on extraction efficiency, process optimization, or antioxidant properties [24,25,26]. In contrast, fewer studies have addressed the full application-oriented pathway linking solvent-dependent phenolic recovery, antiaging-related bioactivity, and direct incorporation of the extract into a cosmetic-compatible matrix. Therefore, the novelty of the present work lies in the integrated evaluation of betaine-based NaDES not only as green extraction media for BP phenolics, but also as formulation-compatible systems for the development of ready-to-use functional ingredients for topical applications.

In this context, the present study evaluated five betaine-based NaDES for the extraction of phenolic compounds from BP, comparing their performance with conventional solvents compatible with food and cosmetic applications (ethanol and water). The obtained extracts were characterized in terms of phenolic content, antioxidant capacity, and anti-enzymatic activity, specifically anti-elastase and anti-tyrosinase activities, to assess their potential suitability as functional ingredients for topical applications. Finally, the most promising extract was incorporated into a natural-based cosmetic formulation, and its main physicochemical properties were evaluated as a preliminary assessment of formulation compatibility.

## 2. Materials and Methods

### 2.1. Plant Material and Sample Preparation

Blueberry (*Vaccinium corymbosum* L.) pomace (BP), generated during the juice-making process, was kindly supplied by Polo Agrifood M.I.A.C S.c.p.a. (Cuneo, Italy) and immediately freeze-dried. Lyophilization was performed using a Telstar LyoQuest system (Terrassa, Spain). The dried material was then stored in plastic bags at −20 °C until use. All samples were used within six months of freeze-drying.

### 2.2. Reagents and Solvents

All solvents and reagents used in this study were of analytical grade. Acetic acid, L(+)-lactic acid, ethylene glycol, glycerol (30° Bé), ethanol (96%), hydrochloric acid (37%), sodium hydroxide, sodium nitrite, potassium chloride, sodium acetate, methanol, and potassium persulfate were purchased from Carlo Erba Reagents (Cornaredo, Italy). LC–MS grade water, acetonitrile, and formic acid were obtained from the same supplier and used for chromatographic and mass spectrometric analyses. Additional reagents, namely citric acid (anhydrous), sodium carbonate (anhydrous), and aluminum chloride hexahydrate, were obtained from Scharlab S.L. (Sentmenat, Spain), whereas iron(III) chloride hexahydrate and Folin−Ciocậlteu phenol reagent were supplied by VWR Chemicals (Radnor, PA, USA). Reference standards gallic acid and rutin were acquired from Sigma-Aldrich (St. Louis, MO, USA), while cyanidin-3-*O*-glucoside was purchased from Extrasynthese (Genay, France). Betaine, 2,4,6-tris(2-pyridyl)-s-triazine (TPTZ), 2,2-azino-bis-3-ethylbenzothiazoline-6-sulfonic acid (ABTS), 2,2-di(4-tert-octylphenyl)-1-picrylhydrazyl (DPPH), and Trolox were purchased from Thermo Fisher Scientific (Waltham, MA, USA). Ingredients employed as gelling and emulsifying agents, including xanthan gum, tapioca starch, algin, and sodium stearoyl lactylate (Instathix^®^), were kindly provided by Safic-Alcan S.p.A. (Lainate, Italy); apricot kernel oil was obtained from Farmalabor (Assago, Italy). All reagents required for the anti-elastase and anti-tyrosinase assays were purchased from Merck (Rome, Italy).

### 2.3. NaDES Synthesis

NaDES were prepared in screw-capped glass bottles using a water bath heated to 80 °C for 30 min at 500 rpm, using appropriate amounts of each compound. All NaDES systems were prepared using a HBA:HBD molar ratio of 1:2, and 20% water was added before extraction to reduce viscosity and improve handling as suggested by previous work [27]. The composition and molar ratios of the prepared NaDES are reported in Table 1.

### 2.4. Extraction Process of Phenolic Compounds

The extractions were performed in triplicate in 50 mL glass bottles that were protected from light. The bottles were placed in a shaker at 50 °C for 2 h with agitation set at 200 rpm as described in our previous work [27]. For each extraction, 1 g of BP was mixed with 15 mL of solvent (1:15 solid–liquid ratio). The resulting extracts were filtered through quantitative filter paper (20–25 μm) under vacuum. The filtrates were then placed in Falcon tubes and stored in a freezer (−20 °C) until analysis. All extracts were analyzed within six months of preparation.

Along with NaDES, H_2_O and 50% (*v*/*v*) and 96% (*v*/*v*) ethanol were used as conventional solvents for comparison in the extraction process.

### 2.5. Phenolic Characterization of Extracts

#### 2.5.1. Total Phenolic, Total Flavonoid, and Total Monomeric Anthocyanin Content Assays

Total phenolic content (TPC) and total flavonoid content (TFC) were determined by colorimetric spectrophotometric assays, following the procedures reported by del Río et al. [28]. TPC was quantified using the Folin–Ciocalteu reaction and calculated from a gallic acid calibration curve. Results were reported as milligrams of gallic acid equivalents per gram of dried blueberry pomace (mg GAE/g BP). TFC was assessed through the aluminum chloride complexation assay, using rutin as the reference standard, and expressed as milligrams of rutin equivalents per gram of dried blueberry pomace (mg RE/g BP).

Total monomeric anthocyanin content (TMAC) was evaluated by the pH-differential spectrophotometric method, according to Ferreira-Santos et al. [29]. Quantification was performed using cyanidin-3-*O*-glucoside as the reference compound, and the results were expressed as milligrams of cyanidin-3-*O*-glucoside equivalents per gram of dried blueberry pomace (mg C3GE/g BP).

#### 2.5.2. Identification and Quantification of Phenolic Compounds by HPLC-ESI-MS/MS

##### Phenolic Acids, Non-Anthocyanin Flavonoids, and Phenolic Aldehydes

Phenolic acids, non-anthocyanin flavonoids and phenolic aldehydes in BP extracts were analyzed by HPLC-ESI-MS/MS using an Agilent 1260 HPLC system (Santa Clara, CA, USA) coupled to an AB SCIEX Triple Quad 3500 mass spectrometer (Marlborough, MA, USA). The analytical conditions were adapted from our previously published method [30]. Chromatographic separation was carried out on a Luna C18 column (150 mm × 2.1 mm, 3 µm; Phenomenex, Torrance, CA, USA), injecting 5 µL of each extract. The mobile phase consisted of water with 0.1% formic acid as solvent A and acetonitrile with 0.1% formic acid as solvent B. Separation was achieved under gradient elution at a flow rate of 0.3 mL/min, starting at 2% B for 4 min, increasing to 20% B from 4 to 7 min, then to 90% B from 7 to 14 min, maintaining 90% B from 14 to 15 min, and returning to 2% B from 15 to 17 min.

Mass spectrometric detection was performed using an electrospray ionization source operated in positive/negative ionization mode with a Turbo V™ interface (Marlborough, MA, USA). The ion spray voltage was set at 4500 V, the source temperature at 400 °C, and nitrogen was used as nebulizer and collision gas. Data acquisition was performed in multiple reaction monitoring (MRM) mode using Analyst 1.6.2 software. Quantification was carried out by injecting authentic phenolic standards under the same analytical conditions, and results were expressed as micrograms per gram of dried blueberry pomace (µg/g BP). Retention times, MRM transitions, calibration curves, and linearity parameters of authentic standards are reported in Table S1.

##### Anthocyanins

To quantify the individual anthocyanin content, samples obtained in Section 2.4 were submitted to a centrifugation process for 10 min at 4 °C (10,000× *g*). After that, 100 μL of each supernatant was collected and diluted to a proper concentration (either 10× or 20×) with formic acid 1% aqueous solution. The diluted samples were then filtered using a hydrophilic PTFE membrane (pore size: 0.22 μm, diameter: 4 mm) (FilterBio^®^, PTFE-L Syringe Filter (Nantong, Jiangsu, China)). Aliquots of 20 μL were then transferred to vials and injected into a Thermo Scientific^®^ Dionex UltiMate 3000 UHPLC, coupled with an Accela^®^ PDA detector (Thermo Fisher^®^, Waltham, MA, USA). For chromatographic separation, the following conditions were used: solvent A, 1% formic acid in water; solvent B, 1% formic acid in acetonitrile. The gradient elution program was as follows: 1–13% B (0–2 min), 13–18% B (2–15 min), 18–18.5% B (15–20 min), 18–18.8% B (20–35 min), and 18.8–100% B (35–45 min). The system was maintained at 100% B for 10 min (45–55 min), followed by re-equilibration under the initial conditions (55–60 min). The stationary phase utilized was a reversed-phase C18 Hypersil GOLD^TM^ VANQUISH (Thermo Scientific, Waltham, MA USA) 150 mm × 2.1 mm column with 1.9 μm particle size, operating at a steady flux of 0.3 mL/min and a working pressure of 330 bar.

Quantification was based on peak integration from the chromatographic analysis, using a standard curve (1) of cyanidin-3-*O*-glucoside (C3G) in μg/mL:

(1) $$Concentration\left(\frac{\mu g}{mL}\;of\;C3G\;eq.\right)=\frac{Area\left(mAU.{min}^{-1}\right)}{8.5563\pm 0.9831}$$

For the identification of the individual anthocyanins, the standard solutions were analysed using a ThermoFisher^®^ LTQ XL iontrap quadrupole coupled to a Thermo^®^ Finnigan Surveyor Plus HPLC System (Thermo Scientific^TM^, San Jose, CA, USA). The chromatographic conditions were the same as for the detection. The detection was carried out at 520 nm using a diode-array detector. An aliquot of 20 μL of each sample was analyzed. Double online detection was done by a photodiode spectrophotometer and mass spectrometry. The mass detector was a ThermoFisher^®^ LTQ XL iontrap quadrupole equipped with an electrospray ionization (ESI) interface. The vaporizer and the capillary voltages were 5 kV and 4 V, respectively. The capillary temperature was set at 300 °C. Nitrogen was used as both the sheath and auxiliary gas at flow rates of 40 and 15 arbitrary units, respectively. Spectra were recorded in positive ion mode between *m*/*z* 150 and 1500. Data were obtained using multiple reaction monitoring (MRM) and processed with Xcalibur^TM^ 4.2 SP1 Software (Thermo Scientific^TM^, San Jose, CA, USA).

### 2.6. Antioxidant Capacity Assays

The antioxidant capacity of the liquid extracts was determined using three methods described by Gullón et al. [31]: the 2,2-di(4-tert-octylphenyl)-1-picrylhydrazyl radical scavenging assay (DPPH), the 2,2-azino-bis-3-ethylbenzothiazoline-6-sulfonic acid radical cation decolorization assay (ABTS), and the ferric reducing antioxidant power assay (FRAP), using Trolox as the standard. Each analysis was performed in triplicate, and results were expressed as milligrams of Trolox equivalents per gram of dried BP (mg TE/g BP).

All NaDES solvents were tested using the same antioxidant assays under identical conditions to check for any intrinsic bioactivity. Negligible activity was observed.

### 2.7. Antiaging Properties

The effects of BP extracts on enzymes involved in skin aging were evaluated by assessing their inhibitory activity against tyrosinase, involved in melanin synthesis, and elastase, responsible for elastin degradation. These enzymes were selected as in vitro targets related to pigmentation regulation and extracellular matrix degradation, respectively. Two extracts were tested: the NaDES-selected extract and the optimal extract obtained using a conventional solvent (ethanol). Anti-elastase activity was determined in vitro using porcine pancreatic elastase, while anti-tyrosinase activity was evaluated against tyrosinase, according to the protocol described by Bari et al. [32].

For anti-elastase (AE) assays, extracts were tested at dilutions of 1:25, 1:50, 1:75, and 1:100. For anti-tyrosinase (AT) assays, the same dilution range was applied together with two additional dilutions (1:500 and 1:1000) [32]. All samples were solubilized in a propylene glycol/purified water mixture (80:20, *w*/*w*) containing 0.5% DMSO.

Epigallocatechin gallate (EGCG) and kojic acid (KA) were used as positive controls for AE and AT assays, respectively, while purified water served as the negative control. Reactions were carried out in 96-well microplates, and absorbance was monitored for 60 min with one measurement per minute at 410 nm for AE and 480 nm for AT using a microplate reader (Victor Nivo, PerkinElmer, Waltham, MA, USA).

Enzyme inhibition was calculated according to the following Equation (2):

(2) $$Inhibition\;\left(\%\right)=\frac{\left(Abs\;control-Abs\;sample\right)}{Abs\;control}\times 100\;$$

where *Abs control* represents the absorbance of the control reaction without extract, and *Abs sample* represents the absorbance of the reaction in the presence of the tested extract. All assays were performed in triplicate.

All NaDES solvents were subjected to the same anti-enzymatic assays, under the same conditions, to verify the absence of intrinsic bioactivity; however, negligible activity was detected.

### 2.8. Preliminary Formulation Study

#### 2.8.1. Preparation of Emulgels

An emulgel formulation was used to incorporate the selected BP NaDES extract. Three emulgels based exclusively on natural ingredients were prepared following the procedure described by Picco et al. [33]: (i) an emulgel containing the selected NaDES extract (Em1), (ii) a control emulgel containing the corresponding NaDES without extract (Em2), and (iii) a blank emulgel prepared with purified water (Em3). The oil phase consisted of apricot kernel oil (2% *w*/*w*); the emulsifying system (3% *w*/*w*) comprised a multifunctional blend of xanthan gum, tapioca starch, algin, and sodium stearoyl lactylate. The aqueous phase consisted of purified water (q.b.) supplemented with either the NaDES extract or NaDES solvent (10% *w*/*w*). Before emulsification, the aqueous phase was heated to 70 °C to facilitate dispersion of the multifunctional blend; then, the oil and aqueous phases were combined and homogenized with an Ultra Turrax^®^ homogenizer (Staufen, Germany) at 11,000 rpm for 5 min. The pH of the formulations was adjusted using sodium hydroxide (2 mol/L) to ensure skin compatibility. All formulations were stored at 4 °C ± 1 °C for a maximum of 24 h before further analysis.

#### 2.8.2. Characterization of the Emulgels: Appearance, pH and Viscosity

The organoleptic properties of the emulgels, including appearance, color, and overall homogeneity, were evaluated by visual inspection. The pH was measured using an LLG-pH Meter 7 (LLG Labware, Meckenheim, Germany). Viscosity (mPa∙s) was determined 24 h after preparation using a rotational viscometer (Brookfield DV-II+ Viscometer (Middleboro, MA, USA)), equipped with a small cup adapter. Measurements were performed at 33.0 ± 0.5 °C with spindle 25 at 20 rpm. An aliquot of each emulgel was placed in a thermostatic jacket to maintain constant temperature. Shear stress (SS) and shear rate (SR) data were simultaneously recorded to construct flow curves. The spindle rotation followed a two-phase program: an ascending ramp from 0.05 to 60 rpm, followed by a descending ramp from 60 to 0.5 rpm.

### 2.9. Statistical Analysis

All statistical analyses and data visualizations were performed using R 4.2.1 (R Foundation for Statistical Computing, Vienna, Austria) and GraphPad Prism 8 (San Diego, CA, USA). Experiments were performed in triplicate, and all data were expressed on a dry weight basis as mean ± standard deviation (SD). Differences were estimated using analysis of variance (ANOVA) followed by Tukey’s honest significant difference test. Differences were considered statistically significant at *p* < 0.05.

## 3. Results and Discussion

### 3.1. Phenolic Content of the Extracts

**Figure 1:**
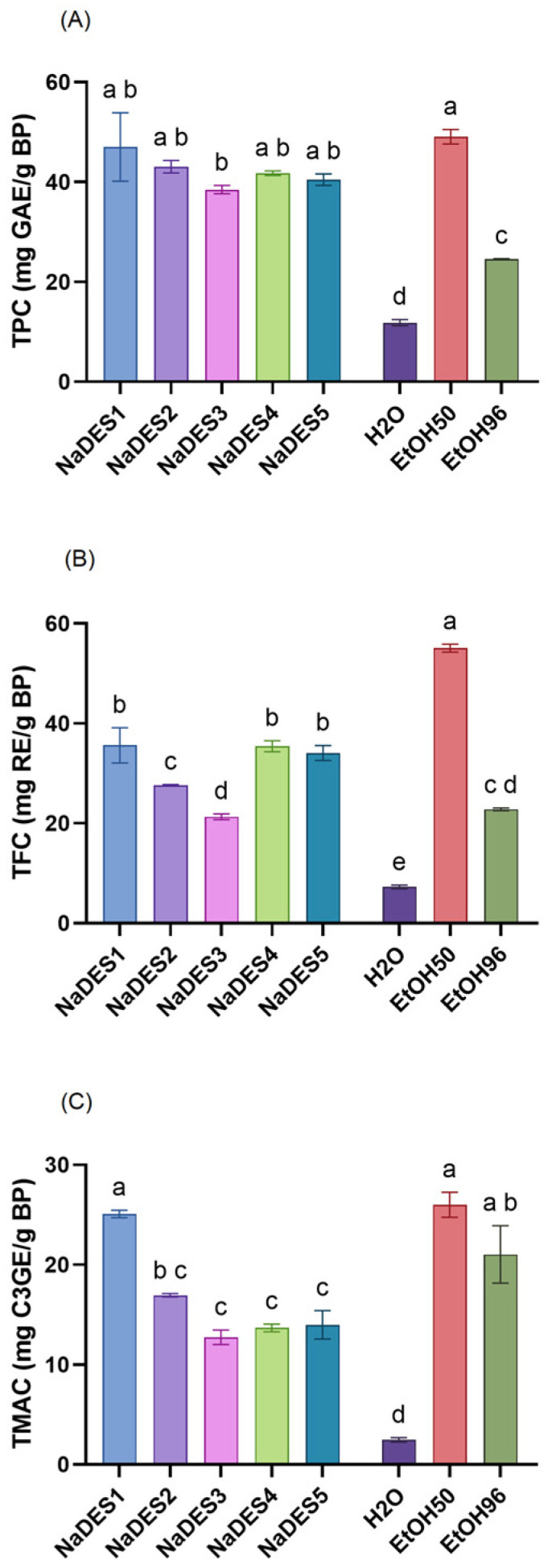
Phenolic content (TPC, TFC, and TMAC assays) in blueberry pomace (BP) extracts expressed as mean ± standard deviation (n = 3). Data are reported on a dry weight basis. Bars with different letters are significantly different (*p* ≤ 0.05). (**A**) TPC, (**B**) TFC, and (**C**) TMAC assays. TPC: total phenolic content, TFC: total flavonoid content, TMAC: total monomeric anthocyanin content, BP: blueberry pomace, GAE: gallic acid equivalent, RE: rutin equivalent, C3GE: cyanidin-3-*O*-glucoside equivalent.

The TPC, TFC, and TMAC of extracts from BP prepared with different NaDES and conventional solvents (water and ethanol at different concentrations) are reported in Figure 1. For TPC, 50% ethanol (EtOH50) yielded the highest phenolic content (49.1 mg GAE/g). This result was not significantly different from those obtained with NaDES1, NaDES2, NaDES4, and NaDES5, which had mean values of 47.0 mg GAE/g, 43.1 mg GAE/g, 41.8 mg GAE/g, and 40.5 mg GAE/g, respectively. NaDES3 had the lowest TPC among NaDES (38.5 mg GAE/g), but it was still significantly higher than the value obtained with EtOH96 (24.6 mg GAE/g) and water (H_2_O, 11.9 mg GAE/g), which exhibited the lowest TPC values. Our results exceeded those reported by He et al. [34], who obtained 16 mg GAE/g following the optimization of ultrasound-assisted extraction, while Dermengiu et al. [35] reported a yield of 32 mg GAE/g from the pomace of Romanian blueberries. Numerous parameters and variables can influence the extraction process, including factors related to the operational procedure, such as the choice of solvent and extraction technique, as well as those associated with the raw material, such as freshness, pre-treatment methods, ripening stage, and cultivar [36,37].

A similar trend was observed for TFC, with EtOH50 achieving the highest flavonoid content (55.1 mg RE/g). Among NaDES BP extracts, NaDES1, NaDES4, and NaDES5 had similar extraction efficiencies (35.6 mg RE/g, 35.4 mg RE/g, and 34.1 mg RE/g, respectively), while NaDES2 and NaDES3 yielded significantly lower flavonoid contents (27.6 mg RE/g and 21.3 mg RE/g, respectively). EtOH96 (22.8 mg RE/g) showed no significant difference from NaDES3, while H_2_O exhibited the lowest TFC (7.26 mg RE/g).

For TMAC, EtOH50 and NaDES1 were the most effective solvents (26.0 mg C3GE/g and 25.1 mg C3GE/g, respectively) and did not differ significantly. EtOH96 showed moderate extraction efficiency (21.0 mg C3GE/g), whereas NaDES2, NaDES5, NaDES4, and NaDES3 formed a group with no significant differences among them but had significantly lower yields than EtOH50 and NaDES1. The lowest anthocyanin content was observed in H_2_O (2.45 mg C3GE/g), further confirming its poor efficiency and highlighting the importance of solvent polarity and bond interaction for phenolic extraction.

While spectrophotometric assays are valuable for preliminary screening, a comprehensive analysis of the phenolic profile requires the precision and specificity of chromatographic methods.

The chromatographic analysis allowed a detailed characterization of the bioactive composition in the extracts. Information from the HPLC-ESI-MS/MS analysis is summarized in Table 2 and Table 3. The extracts show a strong presence of anthocyanins, followed by phenolic acids and non-anthocyanin flavonoids.

This observation aligns with the research conducted by Pertuzatti and colleagues, who reported that anthocyanins in blueberries accounted for more than 70% of the total phenolic content [38,39].

As shown in Table 2, while ethanol-based solvents, particularly EtOH50, demonstrated a high yield (39.1 mg C3GE/g) in extracting total anthocyanins, NaDES have emerged as a sustainable alternative. In detail, among the NaDES extracts evaluated, NaDES1 and NaDES2 exhibited superior extraction efficiencies, yielding 22.2 mg C3GE/g and 21.4 mg C3GE/g, respectively, as total anthocyanins. This improved performance may be related to their higher polarity and enhanced hydrogen-bond donor capacity, which promoted better solubilization and stabilization of anthocyanins. Moreover, the high efficiency of acid-based NaDES containing citric and lactic acids can be attributed to their ability to stabilize anthocyanins under acidic conditions, thereby preserving their structural integrity and bioactivity. This observation is consistent with previous studies that employed acidic NaDES to extract greater quantities of anthocyanins [40,41,42]. In these conditions, anthocyanins primarily exist as flavylium cations, a relatively stable form that is less prone to degradation, thereby enhancing their retention and bioactivity. In contrast, NaDES3, NaDES4, and NaDES5 demonstrated lower extraction yields of 16.2 mg C3GE/g, 11.8 mg C3GE/g, and 10.7 mg C3GE/g, respectively, highlighting the crucial role of NaDES composition in extraction efficacy. Jovanović et al. reported that methanol provides a significantly higher anthocyanin extraction yield from chokeberry (6.99 mg/g) than the most efficient NaDES, a binary mixture of choline chloride and lactic acid, which yielded 5.99 mg/g [43]. These tunable properties of NaDES enable the development of tailored extraction strategies to optimize and maximize anthocyanin recovery.

Overall, despite differences in absolute values likely arising from methodological differences between the analytical techniques, a similar trend was observed for most samples. In terms of anthocyanin profile, 9 compounds were identified, including three malvidin derivatives, three delphinidin derivatives, two petunidin derivatives and a cyanidin derivative (Table 2). All of these have previously been found in BP. The anthocyanins quantified in the extracts were associated with a series of galactoside, glucoside, and arabinoside sugars, following this order in elution that corresponds to the retention behavior of each anthocyanin bound to these sugars. HPLC analysis revealed that malvidin-based anthocyanins were the most abundant across all solvents, reflecting their dominance in BP’s anthocyanin profile.

The anthocyanins derived from methoxylated trisubstituted malvidin were primarily malvidin-3-*O*-galactoside, which emerged as the predominant anthocyanin compound, accounting for 18% in EtOH96 to 26% in NaDES5 of their respective anthocyanin profiles, followed by notable contributions from malvidin-3-*O*-glucoside and malvidin-3-*O*-arabinoside. In terms of quantity, malvidin-3-*O*-galactoside ranged from 7.16 mg C3GE/g in EtOH50 to 0.74 mg C3GE/g in H_2_O, while among NaDES, the highest anthocyanin concentration was achieved in NaDES1, where the binary mixture of betaine and citric acid extracted 4.94 mg C3GE/g. This finding is consistent with recent literature reports, which identified malvidin-3-*O*-galactoside as the most abundant anthocyanin in BP [44,45]. Our findings differ from our previous results, where cyanidin-3-*O*-glucoside was identified as the predominant anthocyanin [46]. These variations can be attributed to multiple factors, primarily differences in blueberry varieties and their geographic origins within the industrial supply chain. In this context, Li et al. analyzed the phenolic profiles of various blueberry varieties harvested in China [47]. They found that certain varieties, such as *Reka* and *Berkeley*, were rich in malvidin-3-*O*-galactoside, while others, like *Sunrise*, contained high levels of delphinidin-3-*O*-galactoside. Petunidin-based anthocyanins, including petunidin-3-*O*-galactoside and petunidin-3-*O*-glucoside, were similarly well-represented in all extracts, accounting for 30.7% of the total anthocyanin composition in EtOH50 and reaching up to 33.6% in NaDES5. These were followed, in order of concentration, by delphinidin-based anthocyanins, which were present as arabinoside, glucoside, and galactoside derivatives. Additionally, cyanidin-3-*O*-glucoside was identified as the only cyanidin-based anthocyanin detected.

A distinct behavior was observed in the extraction of phenolic acids, non-anthocyanin flavonoids and phenolic aldehydes, as quantified by HPLC-ESI-MS/MS, suggesting a nuanced solvent-dependent selectivity for these compounds (Table 3).

In the present study, NaDES demonstrated significant potential for the extraction of phenolic acids and non-anthocyanin flavonoids, with specific formulations yielding higher extraction efficiencies than traditional ethanol-based solvents. Among the tested systems, NaDES3 displayed the highest yield, reaching 208 µg/g, followed closely by NaDES2 (197 µg/g) and NaDES1 (186 µg/g). Statistical analysis indicated that the total amounts extracted with NaDES3 were not significantly different from those obtained with NaDES2, while NaDES2 and NaDES1 showed comparable results. These findings align with those reported by Lončarić et al. (2020), who initially extracted 250 µg/g of dried blueberry pomace before optimizing the extraction process [48]. Ethanol-based solvents, while widely used, displayed a markedly different selectivity profile. The highest yield was obtained with EtOH50 (144 µg/g), which extracted a quantity of phenolics statistically comparable to those obtained with NaDES4 (160 µg/g) and NaDES5 (145 µg/g). As expected, water exhibited the lowest extraction efficiency, reinforcing its limited ability to solubilize polyphenols with diverse structural characteristics, yielding 26.1 µg/g. Water was followed by EtOH96, which extracted a slightly lower amount of 23.9 µg/g, further highlighting the influence of solvent polarity on extraction performance.

The individual composition of phenolic acids and non-anthocyanin flavonoids further reflected the inherent selectivity of NaDES compared to conventional solvents. Chromatographic analysis identified seven phenolic acids (protocatechuic, 4-hydroxybenzoic, ferulic, p-coumaric, vanillic, gallic, and syringic acids), seven non-anthocyanin flavonoids (luteolin, naringenin, galangin, quercetin, rutin, catechin, and epicatechin), and three phenolic aldehydes (vanillin, *p*-hydroxybenzaldehyde, and syringaldehyde). All these compounds have been previously detected in blueberries, both as fruit and by-product, except for galangin [49,50,51], which had the highest concentration in NaDES1, a betaine-citric acid mixture, where it accounted for 1.12 µg/g. This flavonol, known for its documented antimicrobial activity, is primarily found in *Alpinia officinarum* and propolis, and more recently, has also been identified as a minor compound in grapes and red wine [52,53,54,55]. As presented in Table 3, protocatechuic acid was found to be the most abundant compound in all samples, with concentrations varying from 92.4 µg/g in NaDES3 to 4.77 µg/g in EtOH96. Similarly, gallic acid was also identified as a major constituent, following protocatechuic acid, with a notable content of 89.9 µg/g in NaDES2, while its content in EtOH96 was 0.12 µg/g. Other hydroxybenzoic and hydroxycinnamic acids, such as gallic acid and *p*-coumaric acid, were most efficiently recovered using NaDES1, NaDES2, and NaDES3. In contrast, ethanol-based solvents, particularly EtOH50, exhibited a clear preference for flavonoids, as illustrated in the chromatogram in Figure S1, especially for rutin (38.6 µg/g) and epicatechin (10.3 µg/g), indicating that ethanol’s molecular environment is more conducive to the solubilization of these polyphenols.

### 3.2. Antioxidant Activity of the Extracts

**Figure 2:**
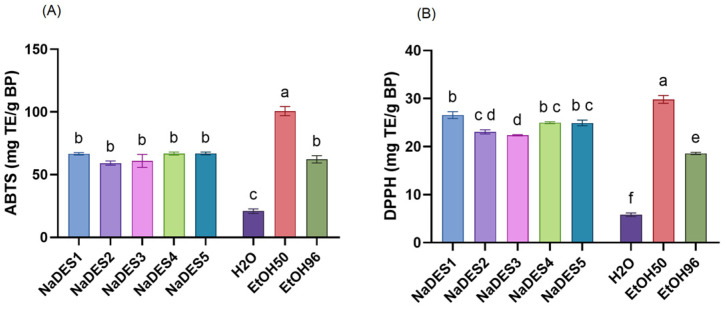
Antioxidant activity (ABTS and DPPH assays) in blueberry pomace (BP) extracts expressed as mean ± standard deviation (n = 3). Data are reported on a dry weight basis. Bars with different letters are significantly different (*p* ≤ 0.05). (**A**) ABTS, (**B**) DPPH assay. TE: Trolox equivalent.

The antioxidant capacity of the BP extracts, evaluated using the ABTS and DPPH radical scavenging assays, revealed solvent-dependent differences that closely mirrored the trends observed for phenolic composition. Both assays operate via electron and hydrogen-atom transfer mechanisms; however, they differ notably in the radical type utilized, solvent compatibility, and their specific sensitivities to classes of antioxidants. While ABTS can function in both aqueous and organic solvents and is sensitive to a broader range of antioxidants, DPPH is primarily restricted to organic solvents and is more selective for certain types of antioxidants. Consequently, these differences result in distinct responses, offering complementary perspectives on the antioxidant behavior of the extracts (Figure 2, Table S2).

Although additional assays based on different antioxidant mechanisms, such as reducing power, can provide useful complementary information, the FRAP assay (Table S3) indicated possible matrix-related interference for acidic NaDES systems, mainly for the citric acid-based formulation and, to a lesser extent, for the acetic and lactic acid-based extracts. This is chemically plausible because FRAP relies on the reduction in the ferric–TPTZ complex and the formation of the Fe^2+^–TPTZ chromophore; therefore, components able to bind or modify iron availability may affect the assay response. Citric acid contains multiple carboxyl groups and can act as a stronger iron-chelating ligand than acetic or lactic acid, potentially competing with TPTZ for iron complexation and leading to an underestimation of reducing power. This interpretation is in line with the findings recently reported by Tsotsou et al., who highlighted matrix-dependent limitations of FRAP-based measurements and suggested that DPPH may be more versatile and less affected by interferents in complex extracts [56]. Therefore, to ensure a consistent comparison among all extracts, the antioxidant evaluation was focused on ABTS and DPPH. Accordingly, the results should be interpreted as comparative radical-scavenging activity rather than as a complete characterization of the antioxidant potential.

In both antioxidant assays, the extract obtained with EtOH50 consistently exhibited the highest radical scavenging activity, confirming its strong ability to recover compounds with pronounced antioxidant capacity from BP, reaching 100 mg TE/g in the ABTS assay and 29.8 mg TE/g in the DPPH assay. This result aligns well with its superior performance in extracting total phenolics. In agreement with our findings, Bamba et al. reported that ethanol concentration strongly affected the antioxidant activity of blueberry pomace extracts obtained by ultrasound-assisted extraction [57]. In their study, 50% ethanol/water was the most efficient solvent system, yielding a DPPH radical scavenging activity of 41.8 mg TE/g dry matter, whereas lower antioxidant activity was observed at less favorable ethanol concentrations, reaching 10.9 mg TE/g dry matter. In the ABTS assay, although EtOH50 stood out with significantly higher activity, no significant differences were observed among the various NaDES extracts, whose values ranged from 66.9 mg TE/g (NaDES5) to 59.2 mg TE/g (NaDES2). A slightly differentiated pattern emerged in the DPPH assay. NaDES1 (26.6 mg TE/g) ranked immediately after EtOH50 and demonstrated the highest scavenging capacity among the NaDES formulations, followed by NaDES4 (25.0 mg TE/g) and NaDES5 (24.9 mg TE/g) extracts.

The antioxidant activity observed in the present study was closely related to the phenolic composition of the extracts; in particular, it depends not only on the total amount of extracted phenolics but also on the specific composition of the extract and possible synergistic interactions among phenolic acids, flavonoids, anthocyanins, and other minor compounds. To further support this interpretation, an exploratory Pearson correlation analysis was performed between HPLC-derived phenolic classes and antioxidant activity. The correlation heatmap is reported in the Supplementary Materials (Figure S2). ABTS activity showed significant positive correlations with total non-anthocyanin flavonoids (r = 0.80, *p* < 0.05) and total anthocyanins (r = 0.77, *p* < 0.05), suggesting that these phenolic classes contributed substantially to the radical-scavenging response measured by this assay. DPPH activity also showed positive correlations with the same phenolic classes, although these associations were not statistically significant. These results support the hypothesis that the antioxidant activity of BP extracts is associated with the combined contribution of different phenolic classes rather than with a single class of compounds. However, due to the limited number of extract systems evaluated, these correlations should be interpreted as exploratory associations rather than evidence of causality. Moreover, the antioxidant activity confirmed the strong influence of solvent type on the recovery of antioxidant compounds from BP. The highest ABTS and DPPH values were obtained for the EtOH50 extract, which was also the most efficient solvent for recovering total phenolics and flavonoids. This result is consistent with previous studies reporting that aqueous ethanol, particularly at intermediate concentrations, improves the extraction of phenolic compounds from blueberry pomace and enhances the antioxidant capacity of the resulting extracts [57]. Indeed, the presence of both water and ethanol can increase the solubilization of phenolics with different polarities, favoring the recovery of anthocyanins, flavonoids, and phenolic acids. In contrast, water alone showed the lowest antioxidant activity, in agreement with its limited extraction efficiency toward several phenolic classes.

Based on the screening results, NaDES1, composed of betaine and citric acid, was selected for further evaluation of its antiaging properties in comparison with EtOH50 and for incorporation into a topical emulgel. Although NaDES2 (betaine:lactic acid) and NaDES3 (betaine:acetic acid) showed comparable or complementary performance in selected phenolic-recovery assays, NaDES1 provided one of the best overall balances among anthocyanin recovery, total phenolic extraction, and antioxidant activity. In addition, the betaine–citric acid system relies on citric acid, which is cost-effective, widely accepted in cosmetic formulations, and more suitable for topical applications than acetic acid because of its better tolerability. EtOH50 was chosen as the best-performing conventional reference solvent, since it provided the highest anthocyanin content, as well as the highest ABTS and DPPH radical-scavenging activities.

### 3.3. Anti-Elastase and Anti-Tyrosinase Activities

The inhibition of elastase and tyrosinase was investigated because these enzymes are involved in two major processes relevant to skin aging and cosmetic applications: extracellular matrix degradation and pigmentation regulation [58,59,60]. Elastase is a proteolytic enzyme involved in the degradation of elastin, a key extracellular matrix protein responsible for skin elasticity and resilience. Increased elastase activity contributes to the loss of dermal elasticity, wrinkle formation and visible signs of photoaging. Therefore, elastase inhibition is commonly considered a useful in vitro indicator of the potential of cosmetic ingredients to protect the extracellular matrix and delay skin-aging-related structural changes. Tyrosinase, on the other hand, is the rate-limiting copper-containing enzyme in melanogenesis, catalyzing the initial oxidation steps leading to melanin formation. Although melanin has an essential photoprotective function, excessive tyrosinase activity and dysregulated melanogenesis are associated with hyperpigmentation conditions such as melasma, solar lentigines and post-inflammatory hyperpigmentation. Consequently, tyrosinase inhibition is widely used as an in vitro marker for evaluating ingredients intended to regulate excessive pigmentation and promote a more uniform skin tone. In this context, the evaluation of both enzymes provides preliminary information on the multifunctional cosmetic potential of the extracts, targeting both loss of elasticity and pigmentation-related alterations.

**Figure 3:**
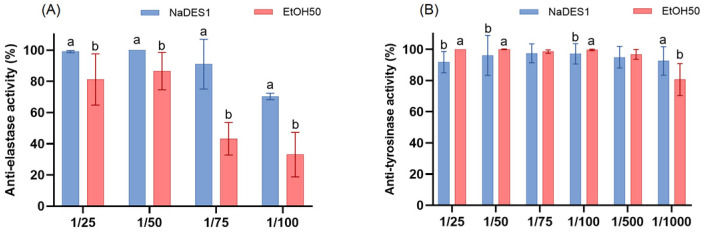
Anti-elastase and anti-tyrosinase activities (%) of NaDES1 and EtOH50 extracts at different dilution ratios. Values are expressed as mean ± standard deviation (n = 3). Bars with different letters indicate statistically significant differences (*p* ≤ 0.05). Where no letters are shown, no statistically significant difference was observed. (**A**) Anti-elastase assay, (**B**) Anti-tyrosinase assay.

The betaine–citric acid NaDES (NaDES1) BP extract was selected and tested for its anti-elastase activity compared to the conventional 50% ethanolic extract (EtOH50). As illustrated in Figure 3A, the NaDES1 extract consistently achieved significantly higher percentages of elastase inhibition at each dilution.

At the highest concentration (1:25), NaDES1 almost completely inhibited elastase activity (99.0%), whereas EtOH50 achieved a similar but significantly lower inhibition (81.2%). A comparable inhibitory behaviour was observed at the 1:50 dilution, with NaDES1 maintaining complete inhibition and EtOH50 showing a reduced effect. Upon further dilution to 1:75, a clear divergence between the two extracts became evident, as NaDES1 retained high inhibitory activity (91.0%), while EtOH50 exhibited a marked decrease (43.2%). Even at the highest dilution (1:100), NaDES1 preserved substantial elastase inhibition (70.3%), whereas EtOH50 showed limited activity (33.0%).

In the tyrosinase inhibition assay (Figure 3B), a concentration-dependent pattern was observed for both extracts. At lower dilution ratios (1:25, 1:50, and 1:75), which corresponded to higher extract concentrations, the EtOH50 extract exhibited slightly greater tyrosinase inhibitory activity than the NaDES1 extract. However, at an intermediate dilution ratio of 1:500, the inhibitory capacities of both extracts were similar, with no statistically significant difference detected. Notably, at the highest dilution (1:1000), NaDES1 retained higher tyrosinase inhibition than EtOH50 (92.5% vs. 80.6%), indicating a better preservation of activity upon dilution.

Differences in extract composition and polyphenol stability likely explain the observed inhibitory patterns. The superior elastase inhibition of the NaDES1 extract is attributable to its higher content of targeted bioactive phenolics, particularly phenolic acids and selected flavonoids, compared with EtOH50. Quantitative analysis of total flavonoids and phenolic acids (Table 3) showed that NaDES1 contained a higher amount (186 µg/g) than EtOH50 (144 µg/g) and was enriched in gallic acid (50.8 µg/g), protocatechuic acid (76.8 µg/g), and luteolin (7.88 µg/g). Together with anthocyanins, these compounds are known inhibitors of matrix-degrading enzymes such as elastase and collagenase, which play a central role in skin aging. Indeed, the degradation of extracellular matrix (ECM) proteins, including elastin, by elastase is a hallmark of extrinsic skin aging and is exacerbated by chronic ultraviolet (UV) exposure [61]. UV-induced oxidative stress promotes the formation of reactive oxygen species (ROS), which activate ECM-degrading enzymes, including metalloproteinases and serine proteases, accelerating ECM breakdown and leading to wrinkle formation, loss of elasticity, and dermal thinning. Consequently, compounds that combine antioxidant activity with inhibition of ECM-degrading enzymes offer a dual-action strategy to mitigate premature skin aging. Flavonoids and phenolic acids, which are abundant in the NaDES1 BP extract, exhibit both radical-scavenging properties and strong inhibitory effects against proteolytic enzymes. This observation is consistent with findings by Mohd Maidin and colleagues [62], who reported that a phenolic-acid-rich hot water extract of red grape pomace showed stronger elastase inhibition than an ethanolic extract richer in flavanols. Gallic acid, in particular, has been shown to interact with elastase through hydrogen bonding and hydrophobic interactions involving its benzene ring and three adjacent hydroxyl groups, thereby interfering with substrate binding. However, the bioactivity of the NaDES1 BP extract cannot be attributed to gallic acid alone. Given the complex and synergistic nature of phytochemical mixtures, the extract should be considered a holistic biochemical matrix rather than a collection of isolated compounds. Cooperative interactions among multiple phenolics, potentially stabilized within the eutectic solvent system, are likely to enhance binding to enzymatic targets. This interpretation is supported by Mohd Maidin and colleagues [62], who observed greater inhibitory activity for whole extracts than for pure gallic acid, and by Wittenauer et al. [15], stronger elastase inhibition by phenolic-rich grape pomace extracts compared with individual purified phenolics at comparable concentrations.

Recent studies on *Vaccinium*-derived matrices further support the suitability of NaDES-based extracts for dermocosmetic applications [63]. For example, NaDES extracts from bilberry fruits and leaves were recently proposed as multifunctional active ingredients for natural dermocosmetic products, showing anti-tyrosinase, anti-hyaluronidase, anti-collagenase, and UV-protective properties. In that study, the anti-tyrosinase activity of the extracts was also evaluated in comparison with kojic acid as a reference inhibitor, confirming the relevance of this enzymatic model for screening depigmenting and antiaging cosmetic ingredients. Although the plant matrix, NaDES composition, and assay conditions differed from those of the present work, these findings support the broader use of NaDES for obtaining phenolic-rich extracts with multifunctional cosmetic potential from *Vaccinium*-derived materials.

Overall, these results indicate that the NaDES1 BP extract acts as a potent elastase inhibitor and a modulator of tyrosinase activity, maintaining bioactivity even at high dilution. By simultaneously targeting oxidative stress and enzyme-mediated ECM degradation, the NaDES1 extract emerges as a promising multifunctional ingredient for topical formulations aimed at reducing UV-induced skin aging, and it was thus selected for formulating emulgels.

### 3.4. Appearance, pH and Viscosity of Emulgels

Emulgels are widely used in topical delivery due to their favorable sensory properties, ease of application, and non-greasy texture. Unlike purely hydrophilic systems such as hydrogels, emulgels reduce the risk of skin dehydration, making them particularly suitable for dry and compromised skin conditions, including xerosis and psoriasis [64].

Evaluating organoleptic and physicochemical properties is essential to assess the suitability of the developed formulations for dermocosmetic application. Before pH correction, the NaDES extract-loaded emulgel (Em1) had a lilac coloration derived from the extract used, changing to yellow after sodium hydroxide was added. The control (Em2) and blank (Em3) emulgels appeared white. All formulations were homogeneous; Em2 and Em3 were odorless, while Em1 had a mild fruit extract scent. The pH of Em1 and Em2 was adjusted to 5.00 ± 0.1, whereas Em3 had an intrinsic physiological pH of 5.40 ± 0.1 and required no adjustment.

Viscosity analysis, performed 24 h after preparation to allow complete structuring of the gelling system, revealed marked differences among the formulations. Em3 was almost fluid, with a viscosity of 6000 mPa∙s, while the NaDES-based emulgels, Em1 and Em2, demonstrated a well-developed network structure and a texture consistent with that of semisolid topical formulations, with viscosity values of approximately 10,800 and 10,400 mPa∙s at 20 rpm, respectively, consistent with dermocosmetic products. This enhanced structuring can be attributed to the intrinsic properties of betaine, which acts as the hydrogen bond acceptor in NaDES1. As a zwitterionic molecule with high water-binding capacity, betaine can function as an electrostatic bridge or cross-linker within the gel matrix, interacting with other formulation components such as tapioca starch in the gelling and emulsifying blend [65]. Similar effects have been reported in topical and polymeric systems, where betaine improves hydration, network formation, and stability. Chen et al. [66] demonstrated that betaine enhanced the performance of collagen-based wound-healing systems by promoting a moist and biocompatible microenvironment, while Liu et al. [67] reported improved gel strength and rheological properties in starch-based matrices through cross-linking interactions involving betaine. Accordingly, Em1 and Em2 exhibited non-Newtonian pseudoplastic behavior with shear-thinning and thixotropic characteristics, as evidenced by their up- and down-flow curves (not reported), whereas Em3 did not. This rheological behavior is particularly advantageous for topical applications, as it ensures formulation stability during storage and packaging while facilitating spreadability and ease of application upon skin contact. The observed behavior can be attributed to the enhanced polymeric network formed by the gelling and emulsifying system in the presence of NaDES. It should be emphasized that this formulation study represents a preliminary compatibility assessment, aimed at verifying the feasibility of incorporating the selected NaDES extract into a natural-based topical matrix. Further stability studies, including accelerated storage, centrifugation, and long-term monitoring, will be required to confirm the technological suitability of the formulation for cosmetic application.

## 4. Conclusions

This study demonstrates that betaine-based NaDES can serve as an effective and sustainable approach for valorizing BP. Through the integration of green extraction strategies, comprehensive phenolic profiling, and functional bioactivity assessment, it was shown that selected NaDES systems, particularly the betaine–citric acid formulation (NaDES1), enable the recovery of phenolic-rich extracts with marked antioxidant properties and significant inhibitory effects on enzymes involved in skin aging. The superior anti-elastase performance and sustained tyrosinase inhibition observed for the NaDES1 extract indicate that solvent design can modulate not only extraction yield but also biological functionality, opening new perspectives for tailoring bioactive profiles. From a sustainability standpoint, this approach supports circular-economy strategies by transforming berry-processing waste into safe, ready-to-use cosmetic ingredients without the need for solvent removal.

Nevertheless, further studies will be necessary to validate these preliminary findings and support the practical application of the selected NaDES extract as a cosmetic ingredient. Future work should focus on the physicochemical properties of the selected NaDES systems and confirming their biological activity using complementary in vitro models, evaluating their safety and skin compatibility, and assessing the long-term stability and performance of the final formulation under relevant storage and use conditions. These additional investigations will help to better define the potential of BP NaDES extracts as multifunctional ingredients for sustainable cosmetic applications.

## Figures and Tables

**Table 1:** Composition of NaDES.

Acronym	Composition HBA:HBD	Molar Ratio	Water Added
NaDES1	Betaine:citric acid (Bet:CA)	1:2	20%
NaDES2	Betaine:lactic acid (Bet:LA)		
NaDES3	Betaine:acetic acid (Bet:AA)		
NaDES4	Betaine:glycerol (Bet:Gly)		
NaDES5	Betaine:ethylene glycol (Bet:EtGly)		

HBA: hydrogen bond acceptor; HBD: hydrogen bond donor. The water content is expressed as % *v*/*v*.

**Table 2:** Individual anthocyanin content (mg C3GE/g dry weight) in BP extracts obtained with NaDES and conventional solvents.

Individual Anthocyanin Compounds	NaDES1	NaDES2	NaDES3	NaDES4	NaDES5	H_2_O	EtOH50	EtOH96
Delphinidin-3-*O*-galactoside	1.46 ± 0.18 ^b^	1.34 ± 0.06 ^b^	0.799 ± 0.030 ^c^	0.407 ± 0.020 ^cd^	0.325 ± 0.070 ^cd^	0.068 ± 0.030 ^d^	3.12 ± 0.15 ^a^	1.66 ± 0.01 ^b^
Delphinidin-3-*O*-glucoside	1.47 ± 0.19 ^bc^	1.49 ± 0.07 ^bc^	1.01 ± 0.03 ^cd^	0.530 ± 0.000 ^de^	0.464 ± 0.08 ^de^	0.162 ± 0.10 ^e^	2.75 ± 0.20 ^a^	1.76 ± 0.01 ^b^
Delphinidin-3-*O*-arabinoside	3.10 ± 0.26 ^c^	3.02 ± 0.12 ^c^	2.16 ± 0.09 ^d^	1.53 ± 0.01 ^de^	1.33 ± 0.12 ^e^	0.33 ± 0.12 ^f^	6.36 ± 0.20 ^a^	4.94 ± 0.02 ^b^
Petunidin-3-*O*-galactoside	4.14 ± 0.43 ^b^	3.90 ± 0.09 ^b^	3.01 ± 0.10 ^bc^	2.35 ± 0.00 ^c^	2.19 ± 0.17 ^c^	0.57 ± 0.27 ^d^	6.48 ± 0.30 ^a^	5.64 ± 0.05 ^a^
Petunidin-3-*O*-glucoside	2.94 ± 0.30 ^b^	3.01 ± 0.10 ^b^	2.22 ± 0.12 ^bc^	1.58 ± 0.00 ^c^	1.41 ± 0.21 ^c^	0.40 ± 0.21 ^d^	5.50 ± 0.15 ^a^	4.60 ± 0.14 ^a^
Cyanidin-3-*O*-glucoside	0.93 ± 0.12 ^bc^	1.05 ± 0.09 ^b^	0.76 ± 0.04 ^bc^	0.56 ± 0.04 ^cd^	0.56 ± 0.03 ^cd^	0.17 ± 0.05 ^d^	2.18 ± 0.09 ^a^	1.79 ± 0.10 ^a^
Malvidin-3-*O*-galactoside	4.94 ± 0.33 ^b^	4.50 ± 0.05 ^bc^	3.72 ± 0.13 ^cd^	2.94 ± 0.03 ^d^	2.80 ± 0.29 ^d^	0.74 ± 0.19 ^e^	7.16 ± 0.04 ^a^	6.55 ± 0.25 ^a^
Malvidin-3-*O*-glucoside	2.10 ± 0.18 ^b^	2.02 ± 0.06 ^b^	1.65 ± 0.10 ^bc^	1.32 ± 0.02 ^cd^	1.05 ± 0.05 ^d^	0.37 ± 0.14 ^e^	3.33 ± 0.09 ^a^	3.19 ± 0.02 ^a^
Malvidin-3-*O*-arabinoside	1.07 ± 0.10 ^b^	1.11 ± 0.08 ^b^	0.89 ± 0.05 ^bc^	0.57 ± 0.01 ^cd^	0.59 ± 0.09 ^cd^	0.22 ± 0.02 ^d^	2.19 ± 0.07 ^a^	1.88 ± 0.05 ^a^
**Total**	**22.2 ± 2.1 ^c^**	**21.4 ± 0.7 ^cd^**	**16.2 ± 0.7 ^de^**	**11.8 ± 0.0 ^e^**	**10.7 ± 1.0 ^e^**	**3.04 ± 1.12 ^f^**	**39.1 ± 0.9 ^a^**	**32.0 ± 0.6 ^b^**

Results are expressed as mean ± standard deviation (n = 3). Different letters within a line indicate statistically significant differences (*p* < 0.05).

**Table 3:** Individual phenolic acids, non-anthocyanin flavonoids and phenolic aldehydes content (µg/g dry weight) in BP extracts obtained with NaDES and conventional solvents.

Individual Phenolic Compounds	NaDES1	NaDES2	NaDES3	NaDES4	NaDES5	H_2_O	EtOH50	EtOH96
**Phenolic acids**								
Protocatechuic acid	76.8 ± 3.3 ^b^	78.3 ± 0.300 ^b^	92.4 ± 3.5 ^a^	69.6 ± 5.55 ^b^	67.9 ± 2.06 ^b^	10.3 ± 0.46 ^d^	46.5 ± 2.78 ^c^	4.77 ± 0.10 ^d^
4-Hydroxybenzoic acid	0.355 ± 0.025 ^abc^	0.417 ± 0.200 ^ab^	0.427 ± 0.030 ^a^	0.313 ± 0.030 ^bc^	0.264 ± 0.020 ^cd^	0.065 ± 0.010 ^ef^	0.168 ± 0.050 ^de^	nd
Ferulic acid	0.69 ± 0.10 ^b^	1.66 ± 0.09 ^a^	1.48 ± 0.07 ^a^	nd	nd	nd	nd	nd
*p*-Coumaric acid	15.9 ± 1.18 ^b^	8.15 ± 0.15 ^c^	15.5 ± 1.12 ^b^	7.90 ± 0.48 ^c^	6.25 ± 0.15 ^cd^	3.68 ± 0.14 ^d^	21.9 ± 1.25 ^a^	5.21 ± 0.320 ^cd^
Vanillic acid	9.41 ± 0.50 ^c^	7.47 ± 0.19 ^d^	9.54 ± 0.46 ^bc^	10.9 ± 0.07 ^b^	13.4 ± 0.5 ^a^	1.52 ± 0.3 ^f^	4.37 ± 0.56 ^e^	0.845 ± 0.110 ^f^
Gallic acid	50.8 ± 6.60 ^bc^	89.8 ± 5.6 ^a^	62.1 ± 0.6 ^b^	49.8 ± 1.70 ^bc^	43.7 ± 2.39 ^c^	5.81 ± 0.65 ^d^	4.68 ± 0.52 ^d^	0.123 ± 0.000 ^d^
Syringic acid	nd	0.479 ± 0.050 ^c^	0.827 ± 0.050 ^b^	1.34 ± 0.11 ^a^	nd	0.064 ± 0.010 ^d^	0.099 ± 0.010 ^d^	0.123 ± 0.010 ^d^
**Flavonoids**								
Galangin	1.12 ± 0.17 ^a^	0.741 ± 0.460 ^abc^	0.596 ± 0.060 ^abcd^	0.813 ± 0.040 ^ab^	0.327 ± 0.060 ^bcd^	0.100 ± 0.020 ^cd^	nd	nd
Luteolin	7.88 ± 0.43 ^a^	3.03 ± 0.60 ^bc^	4.65 ± 0.25 ^b^	6.49 ± 0.53 ^a^	2.97 ± 0.14 ^c^	1.09 ± 0.04 ^d^	2.92 ± 0.71 ^c^	0.716 ± 0.030 ^d^
Naringenin	nd	nd	nd	nd	nd	0.080 ± 0 ^b^	4.16 ± 1.12 ^a^	1.34 ± 0.08 ^b^
Quercetin	nd	0.398 ± 0.160 ^cd^	0.777 ± 0.160 ^bc^	nd	0.082 ± 0 ^cd^	0.144 ± 0.010 ^cd^	3.03 ± 0.45 ^a^	1.36 ± 0.010 ^b^
Rutin	9.69 ± 0.78 ^b^	3.88 ± 0.61 ^cd^	4.28 ± 0.32 ^cd^	2.49 ± 0.11 ^cd^	1.99 ± 0.09 ^d^	1.83 ± 0.29 ^d^	38.6 ± 2.5 ^a^	5.92 ± 0.420 ^bc^
Catechin	0.346 ± 0.053 ^c^	0.361 ± 0.100 ^bc^	0.376 ± 0.030 ^bc^	0.898 ± 0.120 ^b^	0.297 ± 0.010 ^c^	0.101 ± 0.020 ^c^	2.82 ± 0.35 ^a^	0.405 ± 0.010 ^bc^
Epicatechin	5.97 ± 0.49 ^b^	1.21 ± 0.04d ^e^	3.65 ± 0.38 ^c^	1.79 ± 0.01 ^de^	2.13 ± 0.01 ^d^	0.749 ± 0.010 ^e^	10.2 ± 0.51 ^a^	1.92 ± 0.21 ^d^
**Phenolic aldehydes**								
*p*-Hydroxybenzaldehyde	4.63 ± 0.03 ^c^	nd	7.02 ± 0.10 ^a^	6.08 ± 0.39 ^b^	4.94 ± 0.16 ^c^	0.446 ± 0.040 ^e^	2.32 ± 0.27 ^d^	0.308 ± 0.000 ^e^
Vanillin	2.16 ± 0.32 ^b^	0.948 ± 0.110 ^de^	3.44 ± 0.31 ^a^	1.46 ± 0.16 ^cd^	nd	nd	1.92 ± 0.08 ^bc^	0.714 ± 0.100 ^e^
Syringaldehyde	0.370 ± 0.07 ^a^	0.365 ± 0.070 ^a^	0.467 ± 0.040 ^a^	0.406 ± 0.02 ^a^	0.314 ± 0.030 ^a^	0.076 ± 0 ^b^	0.326 ± 0 ^a^	0.085 ± 0.10 ^b^
**Total**	**186 ± 7 ^b^**	**197 ± 4 ^ab^**	**208 ± 3 ^a^**	**160 ± 2 ^c^**	**145 ± 0 ^c^**	**26.1 ± 0.8 ^d^**	**144 ± 2 ^c^**	**23.9 ± 0.6 ^d^**

Results are expressed as mean ± standard deviation (n = 3). Different letters within a line indicate statistically significant differences (*p* < 0.05). nd: not detectable.

## Data Availability

The original contributions presented in this study are included in the article/Supplementary Materials. Further inquiries can be directed to the corresponding author(s).

## Supplementary Materials

The following supporting information can be downloaded at: https://www.mdpi.com/article/10.3390/antiox15060744/s1; Table S1: Mass spectrometric parameters and calibration data for polyphenolic standards analyzed by HPLC-ESI-MS/MS; Table S2: Antioxidant activity (ABTS and DPPH assays) in blueberry pomace (BP) extracts expressed as mean ± standard deviation (n = 3). Data are reported on a dry weight basis. Different letters within the same column indicate statistically significant differences (*p* ≤ 0.05). TE: Trolox equivalent; Table S3: Antioxidant activity (FRAP assay) in blueberry pomace (BP) extracts expressed as mean ± standard deviation (n = 3). Data are reported on a dry weight basis. Different letters within the same column indicate statistically significant differences (*p* ≤ 0.05). TE: Trolox equivalent; Figure S1: Representative extracted ion chromatogram (XIC) of the EtOH50 blueberry pomace extract obtained by HPLC–ESI–MS/MS analysis; Figure S2: Pearson correlation heatmap between HPLC-derived phenolic classes and antioxidant activity of blueberry pomace extracts.

## Abbreviations

The following abbreviations are used in this manuscript: ABTS, 2,2-azino-bis(3-ethylbenzothiazoline-6-sulfonic acid); AE, anti-elastase; AT, anti-tyrosinase; BP, blueberry pomace; C3GE, cyanidin-3-*O*-glucoside equivalents; DES, deep eutectic solvents; DMSO, dimethyl sulfoxide; DPPH, 2,2-di(4-tert-octylphenyl)-1-picrylhydrazyl; ECM, extracellular matrix; EGCG, epigallocatechin gallate; ESI, electrospray ionization; EtOH, ethanol; GAE, gallic acid equivalents; HBA, hydrogen bond acceptor; HBD, hydrogen bond donor; HPLC, high-performance liquid chromatography; LC–MS, liquid chromatography–mass spectrometry; MAPK, mitogen-activated protein kinase; MRM, multiple reaction monitoring; NaDES, natural deep eutectic solvents; NF-κB, nuclear factor kappa-light-chain-enhancer of activated B cells; Nrf2, nuclear factor erythroid 2-related factor 2; RE, rutin equivalents; ROS, reactive oxygen species; SD, standard deviation; SDGs, Sustainable Development Goals; TFC, total flavonoid content; TMAC, total monomeric anthocyanin content; TPC, total phenolic content; TE, Trolox equivalents; TPTZ, 2,4,6-tris(2-pyridyl)-s-triazine; UHPLC, ultra-high-performance liquid chromatography; UV, ultraviolet.
