# Supplementary material for: Green Extraction of Phenolic Compounds from Blueberry (Vaccinium corymbosum L.) By-Products Using Natural Deep Eutectic Solvents for Cosmetic Applications †
Source: Antioxidants (Basel). 2026 Jun 11;15(6):744. doi: 10.3390/antiox15060744 (PMC13296174; doi:10.3390/antiox15060744)
Supplement: Supplementary file 1 [file antioxidants-15-00744-s001.zip › antioxidants-4333769-supplementary.pdf]

SUPPLEMENTARY INFORMATION

Table S1. Mass spectrometric parameters and calibration data for polyphenolic standards analyzed by HPLC-ESI-MS/MS.

Compound name	Rt (min)	Precursor Ion (m/z)	Quantifier Ion (m/z)	Qualifier Ion (m/z)	Ionization Mode	Calibration curve	R^2
Protocatechuic acid	8.20	153	109	—	Negative	y = 6329x-16856.2	0.9995
4-Hydroxybenzoic acid	9.60	137	93	—	Negative	y = 22128x-25810	0.9996
Ferulic acid	11.30	193	178	133	Negative	y = 435.1x+158.9	0.9997
Luteolin	12.39	285	133	—	Negative	y = 729.5x+40.81	0.9996
Naringenin	12.89	271	119	151	Negative	y = 912.1x+2266	0.9992
p-Coumaric acid	11.00	163	119	—	Negative	y = 223.2x+944.4	0.9996
Quercetin	12.40	301	151	121	Negative	y = 4068x	0.9996
Vanillic acid	10.10	167	108	152	Negative	y = 1238x+35.3	0.9995
Vanillin	11.60	151	136	108	Negative	y = 1397x+121.5	0.9998
Syringaldehyde	11.24	183	93	119	Positive	y = 14684x+972000	0.9996
Catechin	9.80	289	244	205	Negative	y = 6292x-4682	0.9999
Epicatechin	10.50	289	245	203	Negative	y = 2684x+2173	0.9999
Syringic acid	10.44	197	181	122	Negative	y = 252.6x+1271	0.9996
Rutin	10.70	609	270	299	Negative	y=661.5x+40.98	0.9991
Gallic acid	4.00	169	124	79	Negative	y=252.6x+1271.4	0.9996

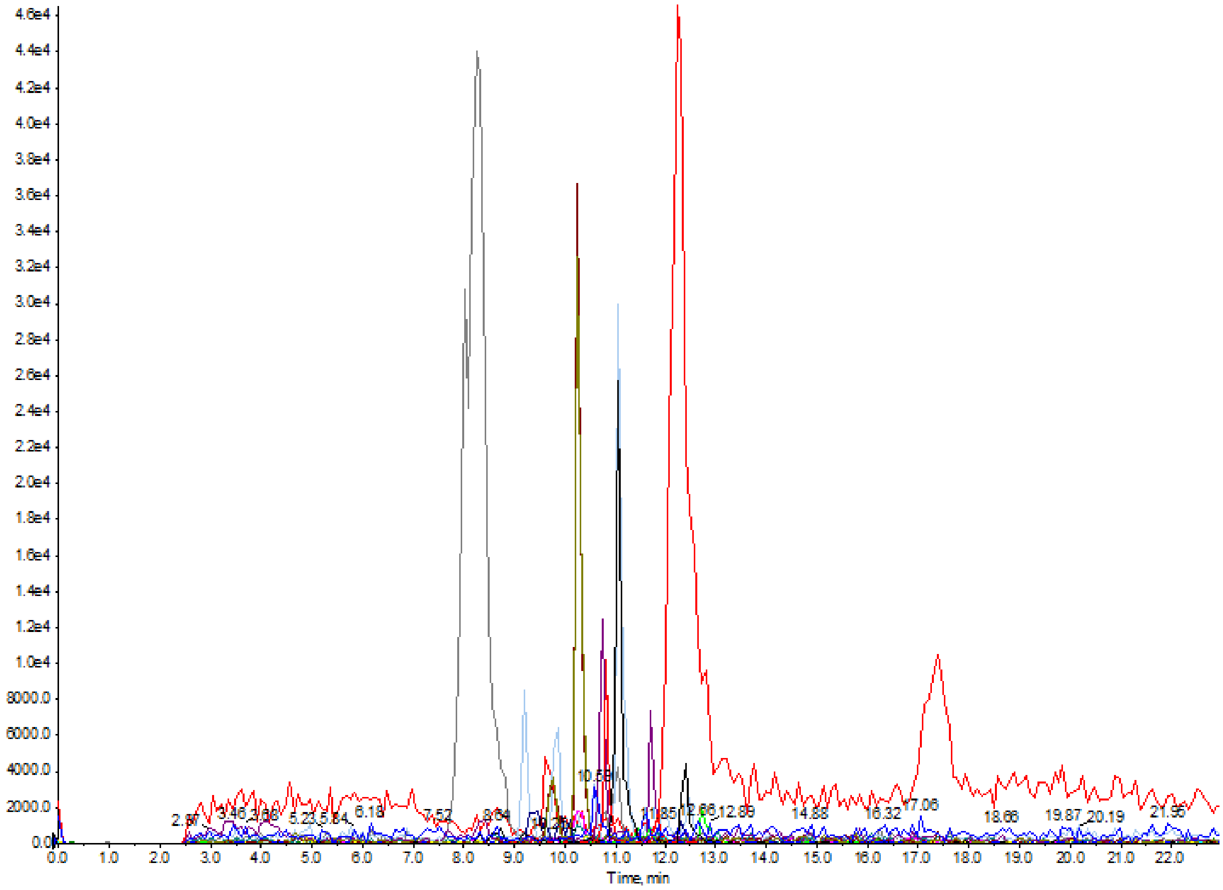

Figure S1. Representative extracted ion chromatogram (XIC) of the EtOH50 blueberry pomace extract obtained by HPLC–ESI–MS/MS analysis.

The chromatogram shows the separation of targeted phenolic acids, non-anthocyanin flavonoids, and phenolic aldehydes. Peak assignment was performed by comparison with authentic standards analyzed under the same chromatographic conditions, considering retention time, precursor ion, quantifier ion, and qualifier ion.

Table S2. Antioxidant activity (ABTS and DPPH assays) in blueberry pomace (BP) extracts expressed as mean ± standard deviation (n = 3). Data are reported on a dry weight basis. Different letters within the same column indicate statistically significant differences (p ≤ 0.05). TE: Trolox equivalent.

**Sample**	**ABTS (mg TE/g)**	**DPPH (mg TE/g)**
NaDES1	66.6 ± 1.0^{b}	26.6 ± 0.7^{b}
NaDES2	59.2 ± 1.7^{b}	23.1 ± 0.4^{cd}
NaDES3	61.0 ± 5.2^{b}	22.4 ± 0.1^{d}
NaDES4	66.9 ± 1.2^{b}	25.0 ± 0.2^{bc}
NaDES5	66.9 ± 1.1^{b}	24.9 ± 0.6^{bc}
H_2O	20.9 ± 1.8^{c}	5.82 ± 0.37^{f}
EtOH50	101 ± 4^{a}	29.8 ± 0.8^{a}
EtOH96	62.3 ± 3.0^{b}	18.6 ± 0.2^{e}

Table S3. Antioxidant activity (FRAP assay) in blueberry pomace (BP) extracts expressed as mean ± standard deviation (n = 3). Data are reported on a dry weight basis. Different letters within the same column indicate statistically significant differences (p ≤ 0.05). TE: Trolox equivalent.

**Sample**	**FRAP (mg TE/g)**
NaDES1	31.9 ± 0.1^{f}
NaDES2	47.6 ± 0.6^{cd}
NaDES3	41.0 ± 1.0^{e}
NaDES4	54.0 ± 1.0^{b}
NaDES5	51.0 ± 1.1^{bc}
H_2O	14.2 ± 1.1^{g}
EtOH50	67.7 ± 3.4^{a}
EtOH96	42.1 ± 0.8^{de}

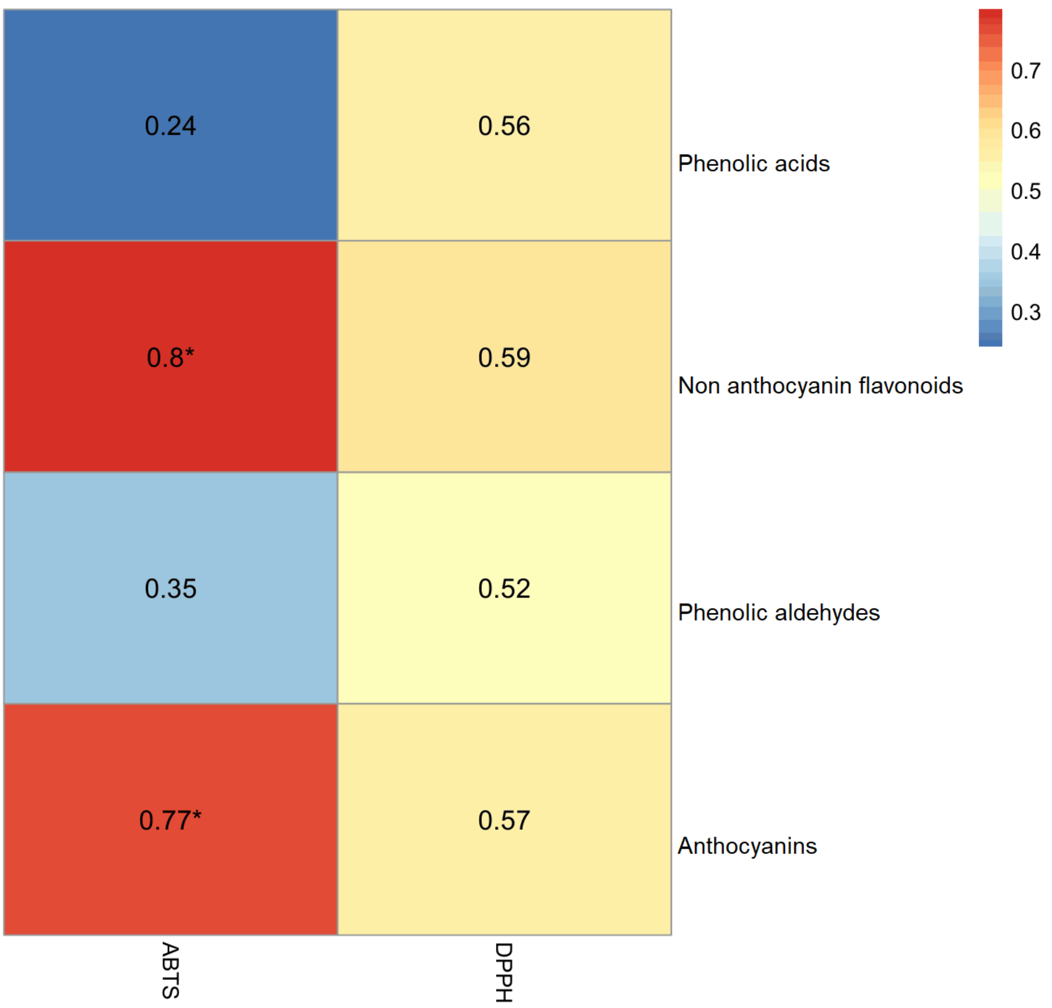

Figure S2. Pearson correlation heatmap between HPLC-derived phenolic classes and antioxidant activity of blueberry pomace extracts. Correlations were calculated using the mean value of each extract as an independent observation (n = 8). Values inside the cells indicate Pearson's correlation coefficients (*r*) between each phenolic class and the antioxidant activity measured by ABTS and DPPH assays. Asterisks indicate statistically significant correlations ($p \leq 0.05$, two-tailed Pearson correlation).
